# I hate you when I am anxious: Anxiety during the COVID‐19 epidemic and ideological hostility

**DOI:** 10.1111/jasp.12914

**Published:** 2022-08-12

**Authors:** Meital Balmas, Tal Orian Harel, Eran Halperin

**Affiliations:** ^1^ Department of Communication The Hebrew University of Jerusalem Jerusalem Israel; ^2^ Department of Communication, The Harry S. Truman Research Institute for Advancement of Peace The Hebrew University of Jerusalem Jerusalem Israel; ^3^ Department of Psychology The Hebrew University of Jerusalem Jerusalem Israel

## Abstract

Most previous studies that examined the effect of anxiety on hostility towards a distinct group have focused on cases in which we hate those we are afraid of. The current study, on the other hand, examines the relationship between anxiety in one domain and hostility towards a distinct group that is not the source of that anxiety. We focus here on symptoms of anxiety during the COVID‐19 pandemic, which have become increasingly frequent, and show that the implications of such mental difficulties are far‐reaching, posing a threat to relationships between ideological groups. In two studies conducted in both Israel and the United States, we found that high levels of anxiety during the COVID‐19 epidemic are associated with higher levels of hatred towards ordinary people from the respective political outgroups, lower levels of willingness to sustain interpersonal relations with these people (i.e., greater social distancing), and greater willingness to socially exclude them. This relationship was mediated by the perception of threat posed by the political outgroup. This study is the first to show that mental difficulty driven by an external threat can be a fundamental factor that explains levels of intergroup hostility.

## 1 INTRODUCTION

The outbreak of the coronavirus (COVID‐19) pandemic occurred in December 2019 in Wuhan, Hubei Province, China, and began to spread throughout China and to the rest of the world in early 2020 (Chen et al., 2020). By January 2021, the coronavirus had infected over 100 million people and claimed the lives of more than two million people worldwide (World Health Organization, 2021). Along with the health crisis, the pandemic has led to a widespread economic crisis as the global economy has acutely contracted and millions of people worldwide are expected to be pushed into extreme poverty (The World Bank, 2020). In times of such crises, societal unity and cohesion help to cope with the threats and preserve stability (Dovidio et al., 2020; Van Bavel et al., 2020a). The current pandemic has posed an even more significant challenge, as it struck at a time when many of the world's democratic countries were dealing with increasing animosity and hostility among political outgroups (Finkel et al., 2020; Reiljan, 2019).

The increasing animosity across party lines (Iyengar et al., 2012) has been one of the world's leading challenges in the recent decade. Numerous studies have indicated that inter‐party hostility is prevalent in many democratic countries (e.g., Gidron et al., 2018; Westwood et al., 2017), and that it has severe implications for both politics (e.g., Hetherington & Rudolph, 2015; Iyengar & Krupenkin, 2018; Ward & Tavits, 2019) and interpersonal relations between supporters of opposing parties (for a review see Iyengar et al., 2019). The growing enmity between ordinary citizens is reflected, among other things, in negative emotions toward supporters of the opposing party—predominantly dislike—and in a tendency to avoid close interactions with them while maintaining social distance (Druckman & Levendusky, 2019; Iyengar et al., 2019). This voluntary detachment impairs societies' resilience and often leads to social and political instability (McCoy et al., 2018).

Therefore, democratic societies around the world are dealing simultaneously both with the COVID‐19 pandemic and with increased hostility across ideological boundaries. The possible links between the COVID‐19 pandemic and the relations between different ideological groups have captured the attention of researchers since the onset of the contagion. Most studies published on these topics have focused on partisan differences in evaluations of the severity of the disease, as well as on differences across party lines in behavioral responses to the pandemic (e.g., Allcott et al., 2020; Druckman et al., 2020, 2021; Grossman et al., 2020; Painter & Qiu, 2020; Pennycook et al., 2020). Both these lines of research point to the politicization of the pandemic (Kerr et al., 2021).

However, when politics are concerned, the COVID‐19 pandemic is not merely another divisive issue on the arena; it also has severe psychological implications that may have further amplified inter‐party animosity. Here we make a case that one such psychological implication of the pandemic, *anxiety*, could have contributed to and perpetuated the already existing hostility. Needless to say, the current study is not the first to examine the anxiety‐hate association. Yet, most existing studies in political psychology examining the effect of anxiety on intergroup hostility have focused on a link within the same domain, such that the same group constituted the source of the anxiety and the target of the hate. For example, individuals from a majority group may fear or be anxious about members of a distinct minority group, and these feelings may trigger hostility or hatred (e.g., Canetti‐Nisim et al., 2008). In that regard, Canetti‐Nisim et al. (2009) found that psychological distress caused by exposure to terrorism predicted perceived threat from Israeli Palestinians, which, in turn, predicted exclusionist attitudes toward this group. Simply put, we hate and wish to exclude those who we are afraid of. In this study, however, we examine the relationship between anxiety triggered by COVID‐19 and hostility towards a distinct political group which is not related to the source of that anxiety. We argue that one plausible mechanism behind this relationship is an increase in sensitivity to threats of different kinds that correlated with anxiety induced by COVID. In other words, it is possible that, compounded with the already existing apprehensions, COVID‐induced anxiety may have a relation with animosity towards outgroups. Previous studies have shown that anxiety leads to an overestimation of threats (Lerner & Keltner, 2000, 2001; Raghunathan & Pham, 1999), and that one of the most pervasive and powerful effects of threat is to increase intolerance and hatred. This relationship is not contingent on whether threat is defined as a widely acknowledged external force or as a subjective, perceived state (Gibson 1998; Marcus et al., 1995; Sullivan, Pierson, & Marcus, 1982). For example, research has uncovered a link between periods of anxiety—economic hard times or work stoppages, for example—and rejection of different outgroups which had little connection with the source of the anxiety (Feldman & Stenner, 1997; Lahav, 2004). In other words, a person who experiences increased anxiety driven by external circumstances may perceive threats of different kinds and feel hatred towards different outgroups whose connection to these perceived threats is indirect at best. Based on previous work (Feldman & Stenner, 1997; Lahav, 2004), we contend that the anxiety triggered by the COVID‐19 pandemic might related the sensitivity to a threat from the political outgroup, which correlated with increased hostility towards members of that group.

## 2 THE COVID‐19 PANDEMIC AND ANXIETY

The coronavirus pandemic poses a threat to people's mental health due to increased and prolonged feelings of fear and uncertainty associated with the virus outbreak (Cao et al., 2020; Ozamiz‐Etxebarria et al., 2020; Torales et al., 2020). A prolonged traumatic event of this kind can reduce people's feelings of security and have adverse effects on their mental health. In the current situation, this impact could be caused by questions related to the pandemic with no definite answers, such as when it will come to an end and what effective methods of treatment exist; constant exposure to a flow of information about the pandemic and its effects; decreased social interactions due to the pandemic; and recommendations, such as remaining at home as much as possible. Symptoms such as anxiety, fear, stress and sleep deprivation have become more frequent during the COVID‐19 pandemic (Cao et al., 2020; Torales et al., 2020). A study conducted in China observed that 53% of people experienced feelings of terror (Zhang & Ma, 2020; and see Wang et al., 2020 for similar data, also from China). In the United States, a cross‐sectional study showed that at least one‐third of young adults reported having clinically elevated levels of depression (43.3%), anxiety (45.4%), and PTSD symptoms (31.8%) (Liu et al., 2020). The rates of anxiety in that study are considerably higher compared to prior studies that have used the same cut points. For example, studies using the GAD‐7 showed the following rates among similar groups: U.S. primary care patients (23.0%; Spitzer et al., 2006), U.S. college students (21.0%; Martin et al., 2014), and U.S. nonveteran community college students (17.4%; Fortney et al., 2016). Similar patterns (i.e., an increase in symptoms such as anxiety, fear and stress due to COVID‐19) were also reported in Australia (Stanton et al., 2020), Cyprus (Salari et al., 2020), Russia (Gritsenko et al., 2020), Spain (González‐Sanguino et al., 2020), Italy (Mazza et al., 2020; Moccia et al., 2020), Iran (Moghanibashi‐Mansourieh, 2020), Turkey (Özdin & Bayrak Özdin, 2020), Denmark (Sønderskov et al., 2020), and Nepal (Samadarshi et al., 2020).

Research on the social and political effects of anxiety indicate that anxious individuals tend to perceive higher levels of risk or threat compared to those experiencing low levels of anxiety (Butler & Mathews, 1983; Eysenck 2013; Lerner & Keltner, 2000, 2001). Not surprisingly, research during COVID‐19 points to a strong relationship between anxiety levels and perceived threat levels regarding the pandemic, i.e., heightened vulnerability or likelihood of contagion (Garfin et al., 2020; Killgore et al., 2020; Lima et al., 2020; Usher et al., 2020). However, as stated above, anxiety is also likely to increase perceived threat in regard to negative events which may not have anything to do with the source of the anxiety (Butler & Mathews 1983, 1987; Huddy et al., 2005). According to Lerner and Keltner (2000, 2001), anxiety produces a sense of uncertainty and lack of control that raises assessments of different threats, whether immediate or remote. Therefore, anxiety during or due to COVID‐19 can, potentially, lead people to anticipate and perceive a variety of threats.

As already stated, we test the hypothesis that such an increase in anxiety will be associated with increased levels of intergroup hostility. As we have already mentioned, such relationships, especially with regard to inter‐party hostility, have thus far been tested mainly within the same domain (i.e., anxiety caused by X relates to hostility towards X). However, in a threatening situation, anxiety may traverse from one domain to another, spreading like contagion (see Lahav, 2004). The domains explored in this study are health and ideology. At the time the study was conducted, the pandemic and the ideological tension were both at their peak (e.g., Finkel et al., 2020), especially in the United States (this issue will be discussed in more detail below, in Study 2). Theoretically, given the increased centrality and saliency of the ideological tension (e.g., Finkel et al., 2020), it may not be too far‐fetched to assume that anxiety triggered by the pandemic would translate into increased hostility and animosity directed at the political outgroup. When ideological tensions are on the rise, the search for a scapegoat in times of anxiety can easily turn the spotlight towards the ideological opponent even though this opponent is not connected to the source of anxiety. Thus, we argue that the mechanism behind the relationship between anxiety and inter‐party hostility is the perceived threat from a political outgroup. That idea has not been tested yet either in the context of inter‐party hostility or during the COVID‐19 pandemic.

In what follows we present two correlational studies. The first one, conducted in Israel provides an initial examination of the relationship between general mental difficulties (levels of anxiety and tension) during COVID‐19 and expressions of (a) hatred towards ordinary people from the political outgroup; (b) willingness to engage in interpersonal relations with members of that political outgroup (i.e., lower social distancing); and (c) willingness to socially exclude those people. In Study 2 we replicate all the results of Study 1, but in the U.S. context and with a larger sample. Furthermore, we introduce in Study 2 a mechanism, perception of threat from the political outgroup, that can potentially explain the associations obtained in the analyses.

## 3 STUDY 1: ANXIETY DURING COVID‐19 AND INTER‐PARTY HOSTILITY; AN INITIAL EXAMINATION IN ISRAELI SOCIETY

The first Study was conducted as a part of a global research project, organized by Van Bavel et al. (2020b), on psychological factors that could be related to responses to the COVID‐19 pandemic. As a part of this project, the team from each country involved was asked to collect data from at least 500 participants in their respective country or territory, representative with respect to gender and age. We should note here that, initially, this project was not designed to include questions about inter‐party hostility. These questions were added only when the poll administered within the project was underway, after approximately 300 subjects had already been sampled. Importantly, the political reality in Israel seemed at that time even more extreme than previously—in the wake of the third round of elections and failed attempts to form a government. Accordingly, and as will be detailed below, the number of respondents for this study was quite small (but still, generally, representative in terms of age, gender, education and income^1^). The small sample is, without a doubt, a limitation that will be discussed in more detail below. Therefore, Study 1 served as an initial examination of the relations between level of anxiety during COVID‐19 and expressions of hostility: emotional (i.e., hatred), interpersonal (i.e., social distance) and socio‐political (i.e., exclusionism). Based on the literature presented above, we hypothesized that higher levels of anxiety and tension would be associated with (a) higher levels of hatred towards ordinary people of the political outgroup, (b) lower levels of desire for interpersonal relations with the people from the political outgroup, and (c) greater willingness to socially exclude those people.

### 3.1 Method

#### 3.1.1 Participants

A total of 167 participants (49.1% female, 50.5% male, and no other categories were found; mean age 46.19, *SD* = 14.59) were recruited, using an online survey platform (The Midgam Panel Project) that offers monetary compensation in return for participation in surveys. Participants were all Jewish Israelis from the general population and the survey was conducted in Hebrew. Based on a sensitivity analysis, we found that our sample of 167 participants afforded 90% power to detect an effect of *f* ^*2*^ = 0.32 size. Education level was measured using 13 values ranging from 1 (upto 8 years of education) to 13 (Doctoral degree) (*M* = 8.17, *SD* = 2.24). Monthly income was measured using 5 values, from (1) *below the average income* to (5) *above the average income* (*M* = 3.07, *SD* = 1.36). Political orientation was measured using 7 values ranging from 1 (*extremely rightwing*) to 7 (*extremely leftwing*) (*M* = 4.08, *SD* = 1.67). The political outgroup set for those who rated themselves on values as 1 (*extremely right‐wing*), 2 (*right‐wing*), and 3 (*moderate right‐wing*) was left‐wing, and the political outgroup set for those who rated themselves on values as 5 (*moderate leftwing*), 6 (*leftwing*) and 7 (*extremely leftwing*) was rightwing. Those who rated themselves on values as 4 (*center*) were presented with a following question: “From which political side do you feel more distant”? The two options were: (1) Right and (2) Left; 57.1% stated they felt more distant from the Right and 42.9% from the Left. The answer to this question set the outgroup for those who defined themselves as “Center.”

#### 3.1.2 Measures

**Level of Anxiety** was measured based on four items. Participants were told: Here are some feelings that one might have due to the outbreak of the coronavirus (COVID‐19) pandemic. For each, please indicate, on a scale of 1 (*not at all*) to 6 (*to a very large extent*), the degree to which you have experienced those feeling (*α* = .92): I have experienced feelings of fear and anxiety; I have experienced stress or tension due to the pandemic situation; I have felt despair and hopelessness; I have felt sadness or a desire to cry. It is important to emphasize that the feelings of tension and anxiety measured here were totally unrelated to the ideological outgroup; namely, we did not ask participants about their fear or anxiety related to the ideological outgroup, but rather, more broadly about their levels of tension, fear and anxiety due to the COVID‐19 pandemic.

**Hatred towards political outgroup**. Participants were asked to indicate, on a scale of 1 (*not at all*) to 6 (*to a very large extent*), to what extent they felt hatred towards ordinary right‐wingers/left‐wingers.

**Desire for interpersonal relations** (i.e., lower social distance) gauges the extent to which individuals are socially comfortable with those on the other political side (Druckman & Levendusky, 2019 *;* Iyengar et al., 2012; Levendusky & Malhotra 2016). We used a set of three questions to capture how comfortable people feel, respectively, having close friends from the other party; having neighbors from the other party; and having their children marry someone from the other party (*α* = .92). Respondents were asked to rate their responses on a scale of 1 (*not at all*) to 6 (*to a very large extent*).

**Social exclusionism of the political outgroup**. Respondents were asked to indicate, on a scale of 1 (*totally disagree*) to 6 (*totally agree*), to what extent they agreed with the following statement regarding their political outgroup: I would prefer to live in a society without right‐wingers/left‐wingers.

##### Covariates

**Socio‐demographic**. We included various socio‐demographic variables that potentially can be related to feelings of anxiety and/or to inter‐party hostility, among them, age, gender, income and level of education. Taylor (2019) noted that COVID‐19 can affect people differently, based on certain sociodemographic factors. This conclusion was corroborated by research. Thus, for example, it was found that women were almost three times more likely to report on feeling of anxiety due to COVID‐19 compared to men (Caycho‐Rodríguez et al., 2021). Younger adults, people with higher education, and people with lower levels of income reported higher anxiety levels than their counterparts with the opposite characteristics (e.g., Lee et al., 2020a; Solomou & Constantinidou, 2020). Those socio‐demographic variables were also found to be relevant for predicting intergroup hostility. For example, Amsalem et al. (2022) found that older adults, people with higher level of education, and women demonstrated greater inter‐party hostility.

**Threat perception due to COVID‐19** has been found closely related to feelings of anxiety during COVID‐19. As stated above, researches have pointed out a strong relationship between anxiety levels during COVID‐19 and perceived threat levels regarding the pandemic, i.e., heightened vulnerability or likelihood of contagion (Garfin et al., 2020; Killgore et al., 2020; Lima et al., 2020; Usher et al., 2020). This variable was measured based on four indicators (e.g., Liu et al., 2021; Van Bavel et al., 2020b): Respondents were asked to indicate, on a scale of 1 (*not at all*) to 6 (*to a very large extent*), to what extent the coronavirus (COVID‐19) threatened their health; the health of their family members; the health of Israelis in general; their personal financial resources; the personal resources of their family; and the Israeli economy in general (*α* = .77).

In addition, to rule out explanations based on factors other than socio‐demographic that the literature mentions as potential predictors of intergroup hostility (Amsalem et al., 2022; Iyengar et al., 2012; Iyengar & Westwood, 2015), we controlled for three variables outlined below:

**Political leaning**. Respondents were asked to indicate their political leaning, on a scale of 1 (*extremely left‐wing/liberal*) to 7 (*extremely right‐wing/conservative*).

**Ideological identity strength**. Previous research has shown that individuals with stronger ideological views and partisan attachments are likely to report higher levels of out‐party animus (Amsalem et al., 2022). This variable was measured based on five indicators (Bankert et al., 2017). Respondents were asked to rate the following items on a scale of 1 (*not at all*) to 6 (*to a very large extent*): How important is your political identity? How well does the term [leftwing/rightwing] describe you? When talking about [leftwing/rightwing], how often do you use “we” instead of “they”? To what extent does your political identity relate to how you define yourself? and To what extent is your political identity strong, compared to your positions on other issues (*α* = .84).

**Moral conviction** was measured based on two indicators (Reifen Tagar et al., 2014; Skitka et al., 2005). Respondents were asked to indicate, on a scale of 1 (*not at all)* to 6 (*to a very large extent*), to what extent their political identity reflected their moral worldview and their beliefs about what is “right” and “wrong” (*α* = .82).

### 3.2 Results

Means, standard deviations, and correlations between the main variables are presented in Table 1. As can be seen, level of anxiety is significantly correlated with hatred and social exclusionism, but not with desire for interpersonal relations (lower social distance). All three dependent variables (e.g., hatred, desire for interpersonal relations and social exclusionism) are correlated with each other in the expected directions. Political identification is positively correlated with desire for interpersonal relations, indicating that, socially, left‐wingers generally feel more comfortable with right‐wingers than vice versa. Not surprisingly, threat perception due to COVID‐19 is significantly and highly correlated with general mental difficulty, which suggests that those who felt more threatened by the consequences of the coronavirus (for both their health and financial resources) reported higher levels of anxiety.

**Table 1:** Means, standard deviations, and inter‐correlations of study variables

	*M*	*SD*	1	2	3	4	5	6	7	8
1. Anxiety	3.44	1.49	1.00							
2. Hatred	1.77	1.18	0.20**	1.00						
3. Desire for interpersonal relations	4.94	1.17	−0.11	−0.38***	1.00					
4. Social exclusionism	2.86	1.79	0.15*	0.33***	−0.38***	1.00				
5. Threat due to COVID	3.87	1.25	0.56***	0.03	−0.08	0.06	1.00			
6. Political identification	4.08	1.67	−0.03	−0.08	0.22**	−0.05	−0.24***	1.00		
7. Ideological identity strength	4.20	1.04	0.03	0.19*	−0.20**	0.19**	0.18*	−0.12	1.00	
8. Moral conviction	4.66	1.01	−0.06	0.03	0.01	0.20**	−0.03	0.28***	0.56***	1.00

*Note*: *p < .05; **p < .01; ***p < .001.

#### 3.2.1 Anxiety during COVID‐19 and intergroup hostility

We ran three separate regressions with hatred, desire for interpersonal relations (i.e., lower social distance) and exclusionism as dependent variables; level of anxiety as an independent variable; and control variables. In line with our initial hypotheses, the analysis presented in Table 2 revealed significant relationships between anxiety during COVID‐19 and a higher level of hatred towards the political outgroup (*b* = .21, *SE* = 0.07, *p* = .006; *f* ^*2*^ = 0.041), lower levels of desire for interpersonal relations (i.e., greater social distancing) with people from the political outgroup (*b* = −.14, *SE* = 0.07, *p* = .04; *f* ^*2*^ = 0.023), and greater desire to socially exclude them (*b* = .33, *SE* = 0.11, *p* = .005; *f* ^*2*^ = 0.039). It should be noted that no interaction effect was found between anxiety and political identification on hatred (*F*_(82)_ = 1.19, *p* = .237), desire for interpersonal relations (*F*_(82)_ = 0.592, *p* = .978), and social exclusionism (*F*_(82)_ = 0.792, *p* = .938). These results suggest that the level of anxiety tested here affects both political sides equally.

**Table 2:** Anxiety during COVID‐19 and expressions of inter‐party hostility

	Hatred	Social distance	Social exclusionism
	*b* (*SE*); *f* ^*2*^	*b* (*SE*); *f* ^*2*^	*b* (*SE*); *f* ^*2*^
**IV**: Anxiety during Covid‐19	.19** (0.07); *f* ^*2*^ = 0.041	−.13* (0.07); *f* ^*2*^ = 0.023	.30** (0.11); *f* ^*2*^ = 0.039
**Controls**:			
Threat perception due to COVID‐19	−.18^a^ (0.09); *f* ^*2*^ = 0.023	.15^a^ (0.08); *f* ^*2*^ = 0.019	−.18 (0.13); *f* ^*2*^ = 0.011
Political identification	−.06 (0.06); *f* ^*2*^ = 0.020	.21*** (0.05); *f* ^*2*^ = 0.000	−.13 (0.09); *f* ^*2*^ = 0.024
Ideological identity strength	.22^a^ (0.11); *f* ^*2*^ = 0.057	−.32** (0.11); *f* ^*2*^ = 0.043	.15 (0.17); *f* ^*2*^ = 0.024
Moral conviction	.00 (0.12); *f* ^*2*^ = 0.007	.00 (0.11); *f* ^*2*^ = 0.009	.40* (0.18); *f* ^*2*^ = 0.001
*R* ^2^	.07**	.17***	.11***

*Note*: Regression models with controlling for demographic measures (age, gender, education, and income) yielded similar results (see Table S1). *p ≤ .05; **p ≤ .01; ***p ≤ .001.

^a^ *p* ≤ .09.

## 4 STUDY 2: THE MEDIATING ROLE OF THREAT PERCEPTION (FROM THE POLITICAL OUTGROUP); EXTENDED REPLICATION IN THE U.S. CONTEXT

The main limitation of the first study is the small sample. In addition to addressing this weakness and replicating the results of Study 1 in a different context (the United States), Study 2 pursued another goal. Study 1 provided evidence for the relationships between anxiety during COVID‐19 and hatred, desire for interpersonal relations and social exclusionism towards the political outgroup. A question that should be asked next is about the possible mechanism behind these relationships. Previous studies pointed at perceived threat from an outgroup (Canetti‐Nisim et al., 2009; Huddy et al., 2005) as a potential mechanism mediating the effects of psychological distress on exclusionist attitudes towards outgroups (see Canetti‐Nisim et al., 2008; Shamir & Sagiv‐Schifter, 2006). However, as noted above, this relationship has mostly been tested in respect to the same domain: psychological distress caused by X relates to hostility towards X, mediated by perceived threat from X. In a highly threating situation, such as the COVID epidemic, anxiety may percolate from one domain to another, spreading like contagion (see Lahav, 2004). Our U.S. study was conducted under extreme socio‐political circumstances: 2 weeks after the storming of the U.S. Capitol, several days before the inauguration of President‐elect Joe Biden, and during the most severe escalation in COVID‐19 casualties, amounting to an average of 3076 deaths per day (a 29% increase over December). It therefore stands to reason that the anxiety due to COVID‐19 could have infected people's perception of a threat from a political outgroup, ultimately augmenting inter‐party hostility.

As noted above, that mechanism has not yet been tested in the context of rivalries between ideological groups, nor in relation to the type of anxiety triggered by global crises such as the current pandemic.

### 4.1 Method

#### 4.1.1 Participants

A total of 757 participants (51.1% female, 48.1% male, 0.4% transgender, and 0.4% with no specific category; mean age 41.60, *SD* = 14.17) were recruited, using Amazon's Mechanical Turk (MTurk)^2^. Participants were all Americans from the general population, and the survey was conducted in English. Since the results of Study 1 pointed to a relatively small effect size (*f* ^*2*^ is between 0.023 and 0.041), in Study 2, we use a larger sample to detect a smaller effect size. Based on a sensitivity analysis, we found that a sample of 757 participants afforded 90% power, and could thus be expected to detect an effect of *f* ^*2*^ = 0.16 size. Education level was measured using four values ranging from 1 (*less than high school*) to 5 (*advanced degree*) (*M* = 3.73, *SD* = 0.90). Monthly income was measured using five values, from (1) less than $30,000 to (5) more than $200,000 (*M* = 2.56, *SD* = 1.10). Regarding political orientation, 48.4% of the respondents defined themselves as Democrats, 40.1% as Republicans, and 11.5% as Independents. The political outgroup set for Democrats was Republicans and vice versa. Those who identified as Independents were presented with the following question: “If you had to choose, do you think of yourself as closer to the Democratic Party or to the Republican Party?” Among this group, 38.6% stated they were closer to Democrats, while 61.8% stated they were closer to Republicans. The answer to this question set the outgroup for those who defined themselves as Independents.

#### 4.1.2 Measures

**Level of Anxiety**. Whereas in Study 1 we measured anxiety as part of general mental difficulties, in this study we searched for a more comprehensive measure of anxiety. To this end, we used the Generalized Anxiety Disorders (GAD 7) inventory (Liu et al., 2020; Spitzer et al., 2006). Participants were told: “We are going to present you with a set of emotions and feelings that you may have felt due to the outbreak of the coronavirus (COVID‐19) pandemic. Please take your time and think how often, over the last 2 weeks, you have experienced the following feelings or emotions.” The options for answers were: (1) not at all, (2) several days, (3) more than half the days, and (4) nearly every day (*α* = .92). The list comprised the following mental states: feeling nervous, anxious or on edge; not being able to stop or control worrying; worrying too much about different things; having trouble relaxing; being so restless that it is hard to sit still; becoming easily annoyed or irritable; and feeling afraid, as if something awful might happen. As mentioned for Study 1, here too, level of anxiety was totally unrelated to the ideological outgroup.

**Hatred towards political outgroup** was measured as specified in Study 1 (on a scale of 1—*not at all* to 7—*very much*. *Desire for interpersonal relations (lower social distance)* (*α* = .93) was measured as specified in Study 1 but on a different scale (running from 0—*not at all comfortable* to 100—*extremely comfortable*), and *Social exclusionism* was measured as specified in Study 1 (on a scale of 1—*totally disagree* to 7—*totally agree*).

**Political intolerance** was included as an additional dependent variable in Study 2, as previous studies had shown that perceptions of outgroup threat can also affect people's inclinations to prevent the outgroup from expressing its positions publicly or from gaining political power and influence—which is tantamount to political intolerance (Gibson & Gouws, 2000). This variable was measured based on three indicators. Respondents were asked to indicate, on a scale of 1 (*totally disagree*) to 6 (*totally agree*), to what extent they agreed with each of the following statements (regarding their political outgroup): I would prefer that Democrats/Republicans be prevented from holding rallies and demonstrations; I would prefer that democrats/republicans be banned from television appearances or speeches; and I would prefer that democrats/republicans not be allowed to visit college campuses to register potential voters (*α* = .94). Since *Social exclusionism* and *Political intolerance* are highly correlated (*α* = .76), and moreover, both gauge a predilection for exclusionist policies, we combined them into one index: *Exclusionist policy*.

**Threat Perception (from the political outgroup)** was measured based on three indicators. Respondents were asked to indicate, on a scale of 1 (*totally disagree*) to 6 (*totally agree*), to what extent they agreed with each of the following statements regarding their respective political outgroup: republicans/democrats are a serious threat to the United States and its people; republicans/democrats endanger the future of the United States; and Republicans/Democrats act in ways that harm American democracy (*α* = .96).

##### Covariates

All covariates were identical to the ones used in Study 1: *Threat perception due to COVID‐19* (*α* = .69) on a scale of 1 (*not at all*) to 5 (*very much*); *Ideological identity strength* (*α* = .91) and *Moral conviction* (*α* = .93) on scale running from 0 (*not at all*) to 100 (*extremely strong*).

### 4.2 Results

Means, standard deviations, and correlations between the main variables are presented in Table 3. As can be clearly seen, anxiety is significantly correlated not only with the three dependent variables (e.g., hatred, desire for interpersonal relations, and exclusionist policy support) in the expected directions, but also with the mediator (e.g., threat from the political outgroup) and the other control variables. Perception of the political outgroup as a threat is highly correlated with all four dependent variables. Additionally, all three dependent variables are correlated with each other. Political identification is correlated with hatred, desire for interpersonal relations, and exclusionist policy support, indicating that Conservatives generally feel more hatred and express more intolerance towards Liberals, and are, socially, less comfortable with Liberals. Here too, threat perception due to COVID‐19 is highly correlated with anxiety.

**Table 3:** Means, standard deviations, and inter‐correlations of study variables

	*M*	*SD*	1	2	3	4	5	6	7	8	9
1. Anxiety	1.87	0.78	1.00								
2. Hatred	3.05	2.03	0.23***	1.00							
3. Desire for interpersonal relations	57.64	28.70	−0.13**	−0.44***	1.00						
4. Exclusionist policy support	3.04	1.75	17***	0.51***	−0.49***	1.00					
5. Threat by the political outgroup	3.90	1.62	0.09**	0.45***	−0.54***	0.63***	1.00				
6. Threat due to COVID‐19	3.73	0.81	0.40***	0.04	−0.04	0.02	0.01	1.00			
7. Political identification	3.95	1.92	−0.12**	−0.08*	0.24***	0.06^a^	0.09**	0.05	1.00		
8. Ideological identity strength	56.29	25.14	0.08**	0.31***	−0.30***	0.29***	0.22***	0.41***	0.10**	1.00	
9. Moral conviction	67.68	25.06	0.07*	0.24***	−0.27***	0.26***	0.13***	0.43***	0.12***	−0.02	1.00

*Note*: *p < .05; **p < .01; ***p < .001.

^a^ *p* ≤ .07.

#### 4.2.1 Anxiety during COVID‐19 and inter‐party hostility in the U.S. context

We replicated the results of Study 1. The analysis presented in Table 4 reveals the relationship between levels of anxiety during COVID‐19 and a higher level of hatred towards people from the political outgroup (*b* = .57, *SE* = 0.10, *p* = .001; *f* ^*2*^ = 0.045), lower levels of willingness to have interpersonal relations with them (i.e., greater social distancing) (*b* = −3.38, *SE* = 1.39, *p* = .005; *f* ^*2*^ = 0.010), and higher levels of support for exclusionist policies (*b* = .35, *SE* = 0.08, *p* = .001; *f* ^*2*^ = 0.026). It should be noted that we found an interaction effect between level of anxiety and political identification on hatred (*F*_(721)_ = 1.37, *p* = .01), which indicates that the relationship between anxiety and hatred is stronger among Republicans than among Democrats. However, no interaction effects were found between level of anxiety and political identification on desire for interpersonal relations (*F*_(721)_ = 1.11, *p* = .11) or exclusionist policy support (*F*_(721)_ = 1.19, *p* = .11).

**Table 4:** Anxiety during COVID‐19 and expressions of inter‐party hostility

	Hatred	Social distance	Exclusionist policy support
	*b* (*SE*); *f^2^*	*b* (*SE*); *f^2^*	*b* (*SE*); *f^2^*
**IV**: Anxiety during COVID‐19	.57*** (0.10); *f* ^*2*^ = 0.045	−3.83** (1.39); *f* ^*2*^ = 0.010	.37*** (0.08); *f* ^*2*^ = 0.026
**Controls**:			
Threat perception due to Covid‐19	−.21* (0.09); *f* ^*2*^ = 0.006	2.53^a^ (1.32); *f* ^*2*^ = 0.004	−.10 (0.08); *f* ^*2*^ = 0.002
Political identification	−.07* (0.03); *f* ^*2*^ = 0.005	3.72*** (0.51); *f* ^*2*^ = 0.067	.05^a^ (0.03); *f* ^*2*^ = 0.004
Ideological identity strength	.02*** (0.00); *f* ^*2*^ = 0.004	−.32*** (0.06); *f* ^*2*^ = 0.005	.01*** (0.00); *f* ^*2*^ = 0.012
Moral conviction	−.00 (0.00); *f* ^*2*^ = 0.000	−.06 (0.06); *f* ^*2*^ = 0.001	.01 (0.00); *f* ^*2*^ = 0.000
*R* ^2^	.14***	.17***	.12***

*Note*: Regression models with controlling for demographic measures (age, gender, education, and income) yielded similar results (see Table S2). *p ≤ .05; **p ≤ .01; ***p ≤ .001.

^a^ *p* ≤ .10.

#### 4.2.2 Mediation analysis

**Figure 1:**
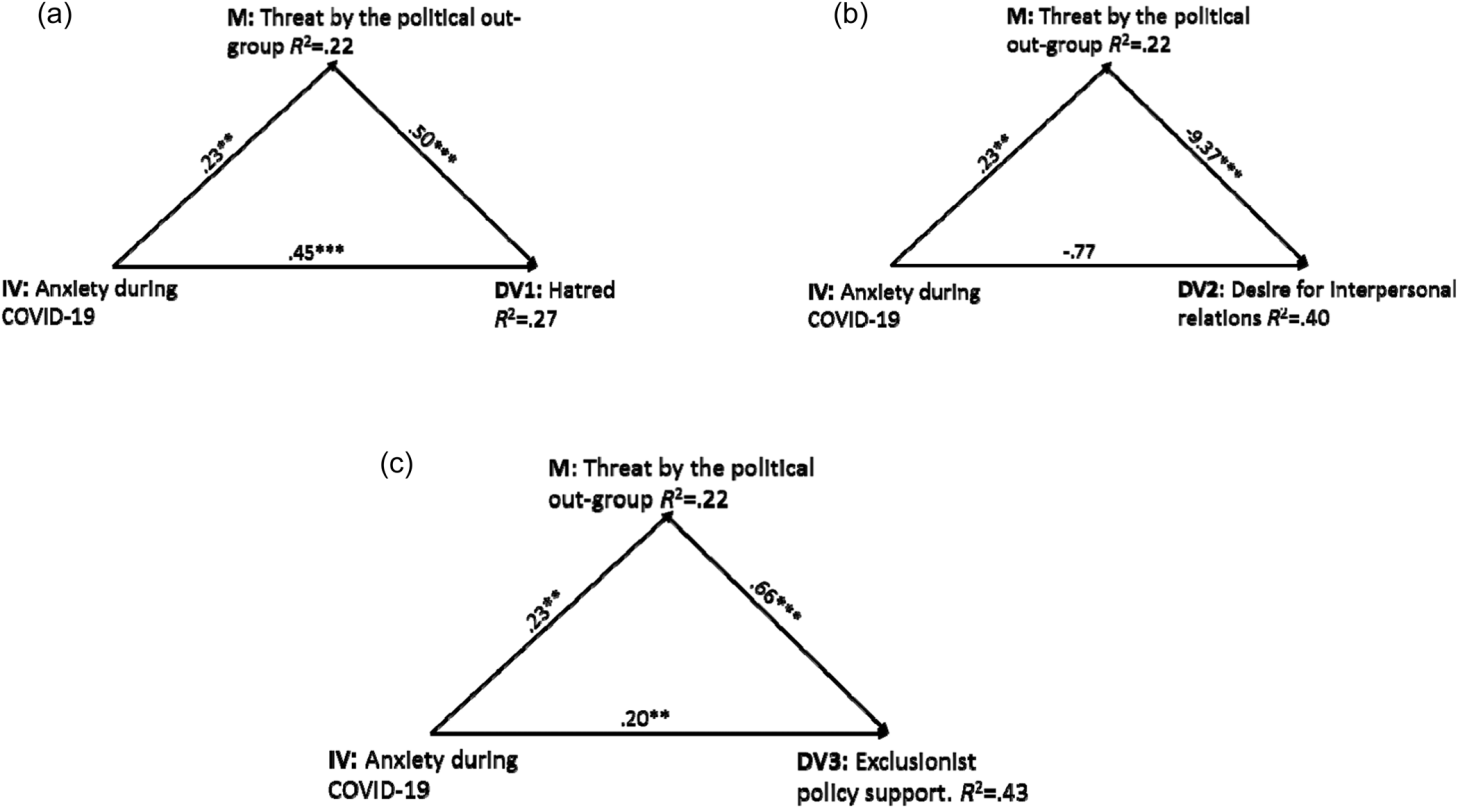
(a–c) Perception of the political outgroup as a threat mediates the association between level of anxiety during COVID‐19 and expressions of inter‐party hostility. (a) Hatred, (b) desire for interpersonal relations, (c) exclusionist policy support. *p < .05; **p < .01; ***p < .001.

We tested whether the links between level of anxiety during COVID‐19 and Americans’ hatred, social distancing tendencies and support for exclusionist policies towards people from the political outgroup were mediated by the perception of the political outgroup as a threat. Figure 1a–c and Table 3 show that level of anxiety significantly increases threat perceptions (*b* = .23, *SE* = 0.07 [0.08, 0.38], *p* = .002; *f* ^*2*^ = 0.008), which relates to a higher level of hatred towards ordinary people from the political outgroup (*b* = .50, *SE* = 0.04 [0.42, 0.59], *p* = .001; *f* ^*2*^ = 0.168), lower levels of willingness to engage in interpersonal relations with them (i.e., greater social distancing) (*b* = −9.37, *SE* = 0.56 [−10.48, −8.25], *p* = .001; *f* ^*2*^ = 0.348); and a higher level of support for exclusionist policies (*b* = .66, *SE* = 0.03 [0.60, 0.73], *p* = .001; *f* ^*2*^ = 0.514). All mediated effects of anxiety on hatred, desire for interpersonal relations, exclusionist policy support, through perception of threat by the political outgroup, while controlling for all control variables, were significant (respectively: *Effect* = 0.11, *SE* = 0.04 [0.03, 0.20]; *Effect* = −2.16, *SE* = 0.74 [−3.60, −0.68]; *Effect* = 0.15, *SE* = 0.05 [0.04, 0.25]) (Table 5).

**Table 5:** Perception of the political outgroup as a threat (republican/democrats) mediates the association between level of anxiety during COVID‐19 and inter‐party hostility expressions

	M: Threat by the political out‐group	DV1: Hatred	DV2: Social distance	DV3: Exclusionist policy support
	*b* (*SE*); *f^2^*	*b* (*SE*); *f^2^*	*b* (*SE*); *f^2^*	*b* (*SE*); *f^2^*
**Main predictors**:				
**IV**: Anxiety during Covid‐19 (GAD 7)	.23** (0.07); *f* ^*2*^ = 0.008	.47*** (0.09); *f* ^*2*^ = 0.037	−.2.11^a^ (1.16); *f* ^*2*^ = 0.004	.24** (0.06); *f* ^*2*^ = 0.017
**M**: Threat by the political out‐group	–	.50*** (0.04); *f* ^*2*^ = 0.168	−9.22*** (0.57); *f* ^*2*^ = 0.348	.66*** (0.03); *f* ^*2*^ = 0.514
**Controls**:				
Threat perception due to Covid‐19	−.10 (0.07); *f* ^*2*^ = 0.000	−.18* (0.08); *f* ^*2*^ = 0.005	2.02^a^ (1.44); *f* ^*2*^ = 0.004	−.07 (0.06); *f* ^*2*^ = 0.001
Political identification	.02 (0.02); *f* ^*2*^ = 0.002	−.09** (0.03); *f* ^*2*^ = 0.009	4.05*** (0.44); *f* ^*2*^ = 0.109	.02 (0.02); *f* ^*2*^ = 0.001
Ideological identity strength	.01** (0.00); *f* ^*2*^ = 0.025	.01*** (0.00); *f* ^*2*^ = 0.027	−.19*** (0.05); *f* ^*2*^ = 0.017	.00** (0.00); *f* ^*2*^ = 0.009
Moral conviction	.01** (0.00); *f* ^*2*^ = 0.032	−.00** (0.00); *f* ^*2*^ = 0.008	.08 (0.05); *f* ^*2*^ = 0.003	−.00* (0.00); *f* ^*2*^ = 0.006
*R* ^2^	.22***	.26***	.38***	.42***

*Note*: Regression models with controlling for demographic measures (age, gender, education and income) yielded similar results (see Table S3). *p ≤ .05; **p ≤ .01; ***p ≤ .001.

^a^ *p* ≤ .09.

## 5 GENERAL DISCUSSION

An extensive body of research published around the world during the past several months of the COVID‐19 pandemic attests to an increase in symptoms of anxiety and fear among the population at large (Cao et al., 2020; Ozamiz‐Etxebarria et al., 2020; Torales et al., 2020). It goes without saying that this change does not bode well for people's mental health. However, its implications are more far‐reaching, posing a threat to the entire social fabric, including relationships between political or ideological groups. In two studies conducted during COVID‐19, one in Israel and the other in the United States, we found that high anxiety levels are associated with higher levels of hatred towards ordinary people from the respective political outgroup, lower levels of willingness to initiate or sustain interpersonal relations with those people (i.e., greater social distance) and greater support for exclusionist policies towards those people. We have also provided evidence that the mechanism behind these relationships is perception of threat posed by the political outgroup. Put differentlly, the perception of threat from the political outgroup is strengthened with the rising anxiety level, leading to hatred, social distancing and exclusionist policy support. Theoretically, we know that anxiety can potentially lead to increased sensitivity to, or overestimation of, threats (Lerner & Keltner 2000, 2001; Raghunathan & Pham 1999). It is also known that threat perceptions can increase hatred, intolerance and exclusionist tendencies towards the source of threat (e.g., Canetti‐Nisim et al., 2008; Gibson 1998; Marcus et al. 1995; Shamir & Sagiv‐Schifter, 2006). However, as already stated, most literature that focuses specifically on inter‐party hostility has thus far explored this relationship within the same domain (i.e., anxiety on account of X relates to perceptions of threat from X and hostility towards the same X). This study examined a mechanism pivoting on perceived threat from the political outgroup during the current pandemic, when the anxiety is driven by an external threat that is largely unrelated (or at least not directly related) to the context of intergroup relations. We concluded that, in the extreme circumstances that the United States were faced with, on account of both the pandemic (i.e., the acute escalation in COVID‐19 casualties) and the socio‐political factors (i.e., 2 weeks after the storming of the U.S. Capitol and several days before the inauguration of President‐elect Joe Biden), anxiety in the health domain might have infected the political domain, increasing people's hostility towards the outgroup. The implication of such mechanism may go beyond the relationship between anxiety due to COVID‐19 and inter‐party relations. Most importantly, it means that times of economic, health, political or other crisis creates a fertile ground for the development of hatred and animosity that go beyond the groups that are perceived as relevant for the creation of the specific crisis (see Lahav, 2004). Societies and leaders, should be aware of these potentially destructive implications, and take some preventive steps to moderate them. Even more broadly, this could mean that people who feel anxious for any reason, e.g., economic difficulties, a physical injury, loss of a loved one and more, tend to regard their environment as replete with threats of various kinds, which may lead them to develop hostility towards a perceived source of the threat. In this sense, the findings presented here can be extrapolated to different anxiety‐triggering situations, different sources of perceived threat, and hostility towards different groups that are thought to pose such a threat. This dynamic, however, requires further research.

From an applied perspective, this is not an easy challenge to address, given that particularly in times of crisis, governmental and societal attention and energy are all funnelled to dealing with the main source of threat (i.e., in the current case—COVID‐19). Yet, increasing social cohesiveness, partly by moderating intergroup hostility, may be one of the most useful tools for dealing with national crisis, and therefore further highlights the importance of addressing the intergroup tensions challenge together with the broader source of national and international crisis. One example for an intervention that mitigated ideological polarization through reducing perceived intergroup threat can be found in the work recently published by Mernyk et al. (2022), in which correcting meta‐perceptions regarding the ideological outgroup's support for intergroup violence decreased participants’ own support for aggressive actions towards that outgroup. These findings were recently replicated in a study conducted during a violent crisis in Israel (Nir et al., 2022), and therefore, provide an interesting example for a path for change, partially aligning with the findings of the current study.

### 5.1 Limitations

This study has several limitations. First, due to its correlational design, we cannot draw conclusions about the *causal* relationship between mental difficulties such as anxiety, on the one hand, and intergroup hostility, social exclusionism and political intolerance, on the other. Establishing the direction of the association demonstrated in the current work would require an experiment that manipulates individuals' anxiety levels, which obviously involves some ethical and moral challenges. Yet, future research can use longitudinal data, relying on enduring measures of mental difficulties or trait anxiety, which can provide additional, albeit not optimal, support to the causal direction intimated by the current study. Second, Study 1, which was conducted in Israel as an initial examination, is based on a community sample that comprises a relatively low number of respondents. Additionally, due to constraints of Study 1, we used there a short and targeted scale of anxiety. However, in Study 2 we use a more comprehensive measure: Generalized Anxiety Disorders (GAD 7) inventory (Liu et al., 2020; Spitzer et al., 2006). Third, our dependent measures are limited to tapping short‐term relationships; this issue can likewise be addressed and elucidated through further investigation.

Last, the studies in the current paper focus on anxiety caused by a specific situational factor, which has so far been (fortunately) very rare: a global pandemic. However, the association between mental difficulties and social exclusionism or political intolerance is probably not limited to this particular context. Therefore, future studies can expand our work and explore this association in other, more common, situations that are considered as conducive to stress and anxiety, for example, poverty. Identifying such situational factors that increase hatred towards ordinary people from the political outgroup can help in future efforts to develop measures to mitigate this phenomenon.

### 5.2 Conclusions

Notwithstanding these weaknesses, the present study provides evidence that the COVID‐19 pandemic has had implications for political intergroup relations. Scholars have argued that one of the leading challenges countries worldwide have faced in the recent decade is the increasing animosity between ideological groups (Druckman et al., 2020; Iyengar et al., 2019). Today, societies around the world need to deal simultaneously with additional threat—the COVID‐19 pandemic. While the current study shows that *mental difficulties* related to COVID‐19 and the threat it poses can contribute to intergroup hostility, an open question that remains to be explored in future studies is whether an extreme challenge, such as a global pandemic, can also have the opposite effect. Can the presence of an external threat suspend the rivalry between ideological groups and encourage them to unite? Can it create the kind of shared goals and identities required to moderate animosity, and under which conditions would that happen? If such a reverse process is indeed possible, the struggle against the pandemic could be channeled into diminishing intergroup hostility and might promote tolerance and respect in the political sphere.

## CONFLICTS OF INTEREST

The authors declare no conflicts of interest.

## ETHICS STATEMENT

Research was conducted ethically, responsibly, and legally. Results are reported clearly, honestly, and without fabrication, falsification or inappropriate data manipulation. New findings are presented in the context of previous research, which is accurately represented. Researchers are willing to make their data available to the editor when requested. Methods are described clearly and unambiguously. Submitted work is original, not (self‐)plagiarised, and has not been published elsewhere. Authorship accurately reflects individuals’ contributions. Funding sources and conflicts of interest are disclosed.

## Supporting information

Supplementary information.

## ENDNOTES

1 For data from the Central Bureau of Israeli Statistics, please see: For age and gender (below board 2.3): https://www.cbs.gov.il/he/publications/Pages/2021/%D7%90%D7%95%D7%9B%D7%9C%D7%95%D7%A1%D7%99%D7%99%D7%94-%D7%A9%D7%A0%D7%AA%D7%95%D7%9F-%D7%A1%D7%98%D7%98%D7%99%D7%A1%D7%98%D7%99-%D7%9C%D7%99%D7%A9%D7%A8%D7%90%D7%9C-2021-%D7%9E%D7%A1%D7%A4%D7%A8-72.aspx For education (below board 1.18): https://www.cbs.gov.il/he/publications/Pages/2021/%D7%A1%D7%A7%D7%A8-%D7%9B%D7%95%D7%97-%D7%90%D7%93%D7%9D-2019.aspx For income: https://www.cbs.gov.il/he/Pages/default.aspx

2 We recruited 771 participants. However, 4 participants failed at two attention check questions (e.g., "This is an attention check. Please select highly agree if you are reading") and 10 participants completed the quaternary in less than 5 min. Accordingly, our final sample included 757 participants.
