# Supplementary material for: I hate you when I am anxious: Anxiety during the COVID‐19 epidemic and ideological hostility
Source: J Appl Soc Psychol. 2022 Aug 12:10.1111/jasp.12914. Online ahead of print. doi: 10.1111/jasp.12914 (PMC9538641; doi:10.1111/jasp.12914)
Supplement: Supplementary file 1 — Supplementary information. [file JASP-9999-0-s001.doc]

**Supplementary Materials for:**

**I hate you when I am anxious: Anxiety during Covid-19 epidemic and ideological hostility**

The three tables presented in this file (S1-S3) are the full regression models controlling for demographic measures. In the article these models are presented without the demographic measures (see Tables 2, 4 and 5 in the article). As can be seen, the models here and in the article yielded similar results.

**Table S1.** Anxiety during Covid-19 and expressions of interparty hostility

|  | Hatred  *b* (*SE*) | Desire for interpersonal relations *b* (*SE*) | Social exclusionism  *b* (*SE*) |
| --- | --- | --- | --- |
| **IV:** Anxiety during COVID-19 | .21**(.07) | -.14*(.07) | .33**(.11) |
| ***Controls:*** |  |  |  |
| Threat perception due to COVID-19 | -.20*(.09) | .14(.08) | -.24~(.14) |
| Political identification | -.05(.06) | .20***(.06) | -.12(.09) |
| Ideological identity strength | .21~(.12) | -.30(.11) | .13(.17) |
| Moral conviction | .03(.12) | -.02(.11) | .43*(.18) |
| ***Socio-demographics:*** |  |  |  |
| Age | -.00(.00) | -.01**(.00) | -.00(.00) |
| Education | -.05(.04) | .01(.04) | -.04(.06) |
| Gender | -.09(.19) | -.11(.17) | -.19(.28) |
| Income | -.02(.05) | .02(.05) | -.08(.08) |
| *R*^2^ | .10*** | .22*** | .14*** |

Note: Regression models without controlling for demographic measures yielded similar results (presented in Table S1). ~p≤0.09, *p≤0.05, **p≤0.01, ***p≤0.001.

**Table S2.** Anxiety during Covid-19 and expressions of hostility and intolerance.

|  | Hatred  *b* (*SE*) | Desire for interpersonal relations *b* (*SE*) | Exclusionist policy support  *b* (*SE*) |
| --- | --- | --- | --- |
| **IV:** Anxiety during the COVID-19 | .57***(.10) | -2.93*(1.39) | .35***(.08) |
| ***Controls:*** |  |  |  |
| Threat perception due to COVID-19 | -.18~(.09) | 2.68*(1.35) | -.12 (.08) |
| Political identification | -.06(.03) | 3.48***(.53) | .05~(.03) |
| Ideological identity strength | .02***(.00) | -.33***(.06) | .01***(.00) |
| Moral conviction | -.00(.00) | -.06(.05) | .01**(.00) |
| ***Socio-demographics:*** |  |  |  |
| Age | -.00(.00) | .11(.07) | -.00(.00) |
| Education | .14~(.08) | -1.88~(1.14) | .10(.07) |
| Gender | -.18(.13) | -4.28*(1.83) | .10(.11) |
| Income | .04(.06) | 1.84*(.94) | -.10~(.05) |
| *R*^2^ | .14*** | .18*** | .13*** |

Note:  ~p≤0.10, *p≤0.05, **p≤0.01, ***p≤0.001.

**Table S3:** Perception of the political out-group as a threat­­­ (Republican/Democrats) mediates the association between level of anxiety during Covid-19 and hostility expressions

|  | ***M***: Threat by the political outgroup  *b* (*SE*) | ***DV1***: Hatred  *b* (*SE*) | ***DV2***: Desire for interpersonal relations  *b* (*SE*) | ***DV3***: Exclusionist policy support  *b* (*SE*) |
| --- | --- | --- | --- | --- |
| ***Main predictors:*** |  |  |  |  |
| ***IV:*** Anxiety during COVID-19 (GAD 7) | .23**(.07) | .45***(.09) | -.77(1.20) | .20**(.07) |
| ***M:*** Threat by the political outgroup | --- | .50***(.04) | -9.37***(.56) | .66***(.03) |
| ***Controls:*** |  |  |  |  |
| Threat perception due to COVID-19 | -.10(.07) | -.13(.09) | 1.74(1.15) | -.05(.06) |
| Political identification | .02(.02) | -.07*(.03) | 3.68***(.45) | .04(.02) |
| Ideological Identity Strength | .01** (.00) | .01***(.00) | -.19***(.05) | .01**(.00) |
| Moral conviction | .01** (.00) | -.00**(.00) | .08(.05) | -.00(.00) |
| ***Socio-demographics:*** |  |  |  |  |
| Age | .01**(.00) | -.00(.00) | .20**(.06) | -.00*(.00) |
| Education | .01(.06) | .13~(.07) | -1.79~(.98) | .10~(.05) |
| Gender | -.02(.10) | -.16(.12) | -4.54**(1.56) | .12(.09) |
| Income | -.08(.05) | .08(.06) | 1.08(.80) | -.05(.04) |
| *R*^2^ | .22*** | .27*** | .40*** | .43*** |

Note:  ~p≤0.10, *p≤0.05, **p≤0.01, ***p≤0.001.
